# In Situ 3D Bioprinting Living Photosynthetic Scaffolds for Autotrophic Wound Healing

**DOI:** 10.34133/2022/9794745

**Published:** 2022-03-20

**Authors:** Xiaocheng Wang, Chaoyu Yang, Yunru Yu, Yuanjin Zhao

**Affiliations:** ^1^Department of Rheumatology and Immunology, Institute of Translational Medicine, The Affiliated Drum Tower Hospital of Nanjing University Medical School, Nanjing 210008, China; ^2^Oujiang Laboratory (Zhejiang Lab for Regenerative Medicine, Vision and Brain Health), Wenzhou, Zhejiang 325001, China; ^3^Wenzhou Institute, University of Chinese Academy of Sciences, Wenzhou 325001, China; ^4^State Key Laboratory of Bioelectronics, School of Biological Science and Medical Engineering, Southeast University, Nanjing 210096, China

## Abstract

Three-dimensional (3D) bioprinting has been extensively explored for tissue repair and regeneration, while the insufficient nutrient and oxygen availability in the printed constructs, as well as the lack of adaptive dimensions and shapes, compromises the overall therapeutic efficacy and limits their further application. Herein, inspired by the natural symbiotic relationship between salamanders and algae, we present novel living photosynthetic scaffolds by using an in situ microfluidic-assisted 3D bioprinting strategy for adapting irregular-shaped wounds and promoting their healing. As the oxygenic photosynthesis unicellular microalga (*Chlorella pyrenoidosa*) was incorporated during 3D printing, the generated scaffolds could produce sustainable oxygen under light illumination, which facilitated the cell proliferation, migration, and differentiation even in hypoxic conditions. Thus, when the living microalgae-laden scaffolds were directly printed into diabetic wounds, they could significantly accelerate the chronic wound closure by alleviating local hypoxia, increasing angiogenesis, and promoting extracellular matrix (ECM) synthesis. These results indicate that the in situ bioprinting of living photosynthetic microalgae offers an effective autotrophic biosystem for promoting wound healing, suggesting a promising therapeutic strategy for diverse tissue engineering applications.

## 1. Introduction

Cutaneous wounds occur for many reasons such as genetic disorders, mechanical trauma, surgical resection, infections, and other diseases [1–5]. Their optimal healing presents a great challenge due to the multifactorial biology and the specific variability of wounds (size, shape, depth, position, duration, patients with diabetic or ischemic diseases, etc.) [6–10]. Extensive and deep defects often necessitate the transplantation of autografts or allografts for wound closure, which may suffer from the shortage of donor tissues, infectious risk, and immunogenic rejection [11–13]. As an alternative and promising option, tissue engineering scaffolds have been elaborated for treating various wounds, especially for the large, deep, and chronic wounds [14–17]. Unfortunately, it is often difficult to fabricate the scaffolds with custom sizes and dimensions, which could adequately cover the wounds with varying depth or topography [18]. In addition, the insufficient nutrient and oxygen availability, particularly in the central regions of the macroscale scaffolds, compromises the overall therapeutic efficacy and results in poor regenerative outcomes [19–21]. Although many oxygen-delivery stratagems through incorporating inorganic peroxides, liquid peroxides, or fluorocarbons into the scaffolds have been made to alleviate systemic hypoxia, promote angiogenesis, enhance collagen remodeling, and accelerate wound closure, these oxygen-generating systems typically provide oxygen for several days and cannot sustain sufficient oxygen over the entire healing process [22, 23]. Therefore, the development of an innovative scaffold with self-adaptive topography and sustained oxygen supply throughout the healing process of different wounds is still highly anticipated.

**Figure 1:**
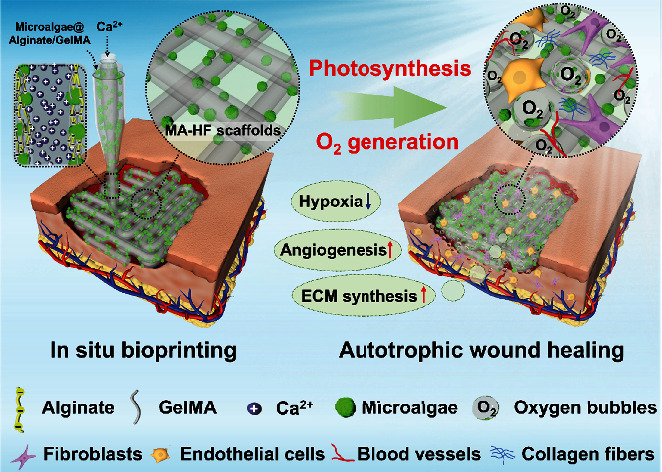
Illustration of in situ 3D bioprinting living photosynthetic scaffolds for autotrophic wound healing. The microalgae-laden hollow fibrous (MA-HF) scaffolds can be directly printed in freeform wounds due to the rapid crosslinking between the Ca ions and alginate-based progels during a coaxial microfluidic printing process. After printing, the microalgae encapsulated in the MA-HF scaffolds serve as in situ autotrophic oxygen suppliers, which continuously generate oxygen under light illumination for enhanced wound healing by alleviating local hypoxia, accelerating angiogenesis, and promoting extracellular matrix (ECM) synthesis at wound sites.

In this paper, inspired by the natural symbiotic relationship between salamanders and algae [24], we present a novel living photosynthetic scaffold by using an in situ three-dimensional (3D) bioprinting strategy for autotrophic wound healing, as schemed in Figure 1. As a primitive autotrophic microorganism, microalga undergoes oxygenic photosynthesis to convert carbon dioxide and water to carbohydrates and oxygen under light illumination [25]. When in a long-term symbiotic relationship with spotted salamanders, the alive algae inside the salamander cells feed on the released waste materials and then photosynthesize to produce oxygen to the host cells [24]. Benefiting from the oxygen-releasing capacity, together with their abundant bioactive components and excellent biocompatibility, the photosynthetic microalgae have recently been incorporated into many functional systems for biomedical applications [20, 26–28]. Additionally, owing to the design flexibility and anatomical accuracy, 3D bioprinting technology has been widely employed for the fabrication of both living cell-laden and acellular biomimetic constructs in tissue engineering [29–34]. In particular, some attempts have been performed by in situ bioprinting of tissue constructs at defect sites, which enables accurate filling of irregular-shaped defects and concurrence of in vivo integration with native tissues [35–40]. Thus, it is conceivable that the in situ bioprinting of the photosynthetic microalgae into wound sites would provide a self-adaptive platform with an autotrophic oxygen supply for versatile wound healing.

Herein, we employed a microfluidic-assisted bioprinting strategy to directly deposit the living microalgae-laden hollow fibrous (MA-HF) scaffolds into the defect sites for promoting wound closure (Figure 1). Microfluidic technology manipulates single or multiple fluids in microscale channels in the range of tens to hundreds of microns [41–43]. The integration of microfluidic systems with conventional 3D printing platforms enables the precise control of the compositional and structural properties of tissue engineering scaffolds during the printing process [44, 45]. Thus, in this study, the microalgae-laden hollow fibers were firstly generated by rapidly polymerizing the microalgae-laden fluid from a coaxial capillary microfluidic chip and then printed into the 3D scaffolds conforming to the irregular-shaped wounds. Owing to the oxygenic photosynthesis of the embedded living microalgae (*Chlorella pyrenoidosa*, one of the most cultivated unicellular microalgae), the scaffolds could produce sustainable oxygen under light illumination, which facilitated the cell proliferation, migration, and differentiation even in hypoxic conditions. It was demonstrated that when the living microalgae-laden scaffolds were directly printed into the diabetic chronic wounds and exposed to light illumination, they could significantly accelerate the wound closure by alleviating local hypoxia, increasing angiogenesis, and promoting extracellular matrix (ECM) synthesis. These results indicated that such living photosynthetic scaffolds with autotrophic oxygen-supply capacity could be promising candidates for wound healing and various tissue engineering applications.

## 2. Results and Discussion

In the present study, the living microalgae-laden hollow fibrous (MA-HF) scaffolds were fabricated by a microfluidic-assisted bioprinting method (Figure 1). Firstly, a coaxial capillary microfluidic chip was custom-made by coaxially inserting a spindle capillary (orifice diameter: 100 *μ*m) into a tapered injection capillary (orifice diameter: 450 *μ*m, Figure S1). The gelatin inner fluid (5% *w*/*v*) containing various CaCl_2_ contents (0~2% *w*/*v*) was pumped into the inner spindle capillary. The mixture fluid of alginate (2.5% *w*/*v*) and GelMA (5% *w*/*v*) biopolymers was pumped into the outer tapered capillary. The hollow fibers were generated by first ionic crosslinking between the Ca ions and alginate biopolymers and subsequent photopolymerization of GelMA components under UV irradiation. The channel diameter of the hollow fibers could be controlled from 150 to 350 *μ*m by changing the CaCl_2_ contents in the range of 0.2~2.0% and the inner/outer flow rate in the range of 0.2~2.0 (Figure S2). Comparatively, an increase in the CaCl_2_ contents led to a decrease in channel diameter, while an increased flow ratio resulted in an increased channel diameter. When the CaCl_2_ contents and flow ratio were fixed as 0.8% and 1.0, a typical hollow fiber with a straight channel was obtained, whose outer and inner diameters were approximately 375 *μ*m and 300 *μ*m, respectively (Figure S3). By introducing the green microalgae (*Chlorella pyrenoidosa*) into the biopolymer matrix, the living microalgae-laden hollow fibers were generated and appeared light green, different from the colorless acellular fibers (Figure S4).

**Figure 2:**
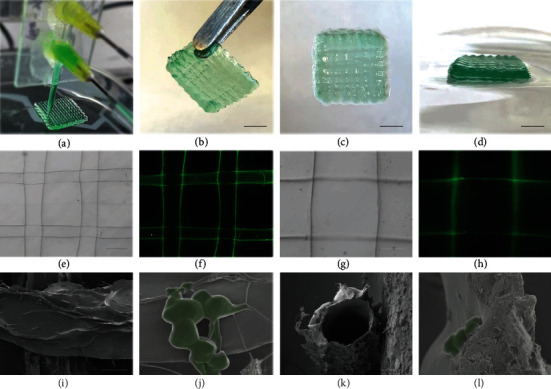
Characterization of MA-HF scaffolds. Photographs of (a) in situ microfluidic bioprinting process and (b–d) different views of the printed hollow fibrous scaffolds containing green dyes. (e–h) Optical and fluorescent micrographs of the hollow scaffolds with straight channels. Green: green fluorescent nanoparticles of 501/515 nm. (i, j) Top and (k, l) sectional views of the scanning electron microscopy (SEM) images of the freeze-dried MA-HF scaffolds at different magnifications. The microalgae encapsulated in the MA-HF scaffolds were shown in pseudogreen (j, l). The scale bars indicate 5 mm in (b–d), 300 *μ*m in (e, f), 100 *μ*m in (g, h, i, k), and 5 *μ*m in (j, l), respectively.

Subsequently, the coaxial microfluidic chip was used to replace the original printing head in a programmable 3D printer. The microfluidic-spun microfibers could be stacked into a 3D scaffold layer by layer on a dry petri dish (Figure 2(a)), when the flow rate of the fluids well matched the moving speed of the printing head. As shown in Figures 2(b)–2(d), the in situ printed constructs could be picked up with tweezers and maintained their shapes in the air. Because the diffusion rate of Ca ions in biopolymer fluid was slower than the printing speed, the solidification of the microfiber was incomplete when the fibers were layer-stacked into a 3D construct, contributing to the good connections of the fibrous struts with straight channels (Figures 2(e)–2(h)). Additionally, the programmable in situ printing platform also enabled feasible control over the dimensions and shapes of the 3D scaffolds (e.g., triangle, cubic, and cylinder constructs in Figure S5), indicating the potential application of in situ printing of living microalgae-laden scaffolds for repairing irregular-shaped defects. The microstructure of the obtained microalgae-laden hollow fibrous (MA-HF) scaffolds was visualized under a scanning electron microscope (SEM, Figures 2(i)–2(l) and Figure S6). The strut surface of the MA-HF scaffold was rougher and more wrinkled, in contrast to the smooth and flat surface of the acellular hollow fibrous (HF) scaffold (Figure S7). The microalgae (a spherical shape of 2~5 *μ*m, Figure S8) were wrapped in hydrogels, distributed randomly on the scaffold surface or embedded in the scaffold struts, confirming the successful incorporation of microalgae into the scaffold matrix. Altogether, the above results demonstrated the feasibility of the in situ bioprinting of living microalgae-laden scaffolds using the microfluidic-assisted bioprinting strategy.

**Figure 3:**
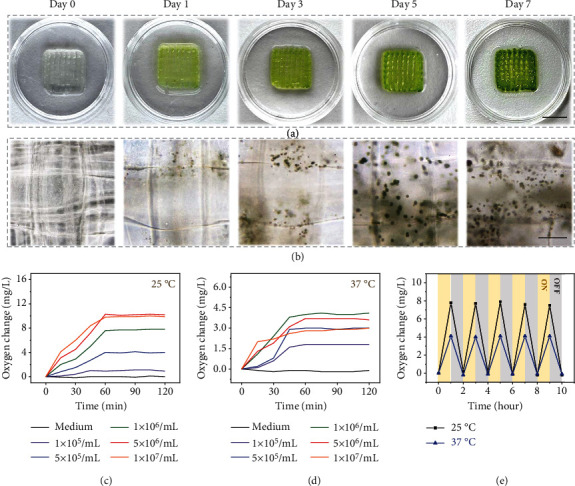
Photosynthetic oxygen-generating capability of MA-HF scaffolds. (a) Digital photographs and (b) bright-field microscopic images of MA-HF scaffolds cultivated for 7 days. (c, d) Quantification of oxygen changes in different MA-HF scaffolds with microalgae concentrations ranging from 10^5^ to 10^7^/mL at (c) 25°C and (d) 37°C. (e) Comparison of oxygen releasing of MA-HF scaffolds under light (ON, yellow) and dark (OFF, gray) conditions at 25°C and 37°C. The scale bars indicate 1 cm in (a) and 150 *μ*m in (b).

Whereas the MA-HF scaffold was almost colorless after in situ printing, it gradually turned green when cultivated at 25°C under continuous light illumination (6000 lux) for 7 days (Figure 3(a)). Accordingly, the bright-field microscopic images revealed a distinct increase in the cell amounts over the culture period (Figure 3(b)), confirming that the microalgae survived from the in situ bioprinting process and maintained their proliferative ability within the scaffolds. To investigate the photosynthetic oxygenation activity of MA-HF scaffolds, the dissolved oxygen (DO) in the medium was monitored using an oxygen microsensor. A rapid increase in DO concentration could be detected in the first 60 min and then reached equilibrium under continuous light illumination, demonstrating the efficient oxygenation capacity of the photosynthetic microalgae encapsulated in MA-HF scaffolds (Figures 3(c) and 3(d)). Quantificationally, the increased DO concentration after 60 min illumination was 0.9, 4.0, 7.8, 10.2, and 9.9 mg/L at 25°C with microalga contents of 1 × 10^5^, 5 × 10^5^, 1 × 10^6^, 5 × 10^6^, and 1 × 10^7^ cells/mL, respectively. The amount of oxygen released from MA-HF scaffolds was reduced when the microalga content exceeded 5 × 10^6^, and a similar phenomenon also appeared when the microalga content was above 1 × 10^6^ at 37°C, indicating that the incorporation of too many microalgae in the scaffold may not be favorable for their growth and metabolism. The oxygen production at 37°C was inferior to that at 25°C, probably because the mammalian-cell culture condition might not be favorable for the microalga cells. With consideration of further wound healing applications, we chose an optimal microalga content of 1 × 10^6^ cells/mL for subsequent studies. Notably, this oxygen-releasing behavior of MA-HF scaffolds was quite sensitive to the light/dark conditions, with an essentially constant increase in DO concentration of ~7.5 mg/L at 25°C and ~4.1 mg/L at 37°C, over five 60 min on/off cycles of light illumination (Figure 3(e)). Additionally, the photosynthetic oxygenation ability of MA-HF scaffolds was not significantly attenuated after storage at 4°C for 7 days (Figure S9). Collectively, all the above results confirmed that the encapsulated living microalgae in the MA-HF scaffolds endowed them with an effective and controllable oxygenation capacity by photosynthesis. The degradation behavior of MA-HF scaffolds was investigated after being soaked in phosphate-buffered saline (PBS, pH 7.4) for a certain period (Figure S10). It was found that the scaffolds lost their original shapes and mechanical strengths when immersed in PBS for 24 h, and a mass loss of 85% was obtained after 5 days. Notably, the microalgae within the MA-HF scaffolds did not grow and gradually degraded in dark conditions. These results indicated that the MA-HF scaffolds are degradable in a physiological microenvironment, which is desirable for biomedical applications including tissue regeneration and wound healing.

**Figure 4:**
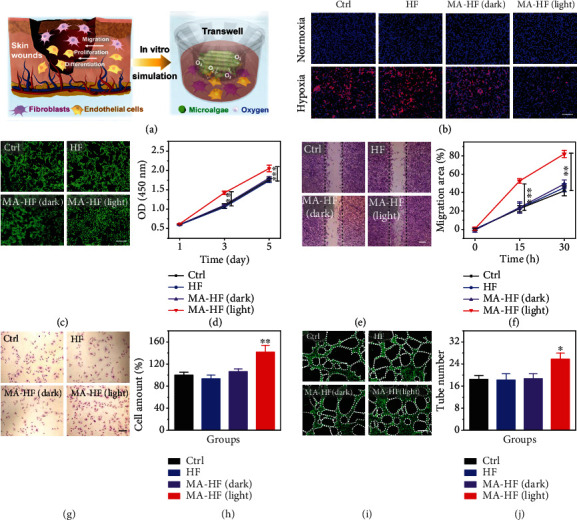
In vitro hypoxia alleviation by MA-HF scaffolds. (a) Schematic illustration of the in vivo skin wound healing process and in vitro experimental design for MA-HF scaffolds. (b) Fluorescent images of human skin fibroblasts (HSFs) incubated in normoxia or hypoxia (1% O_2_) and double stained with hypoxia probe ([Ru(dpp)_3_]Cl_2_, red) and DAPI (blue). (c, d) Representative images and quantification of HSF cultured with HF or MA-HF scaffolds under hypoxic conditions (1% O_2_) with or without light irradiation (6000 lux, 6 hours) for 5 days. (e, f) Representative images and quantification of in vitro scratch assay of HSFs. Dotted lines indicate initial scratch edges. (g, h) Representative images and quantitative analysis of transwell migration assay of human umbilical vein endothelial cells (HUVECs). (i, j) Representative images and quantification of tube formation of hypoxic HUVECs. Dotted circles indicate formed tubes. All scale bars indicate 200 *μ*m. ^∗∗^*p* < 0.01 and ^∗∗∗^*p* < 0.001 compared with control.

Wound healing is an intricate and well-organized process that involves a sequence of events including the proliferation, migration, and differentiation of multiple cells such as fibroblasts and endothelial cells [5] (Figure 4(a)). Oxygen is required in every stage of the wound healing process, and hypoxia (i.e., a lack of oxygen supply) is the most common reason for impaired wound healing [22]. In our study, the MA-HF scaffolds are expected to release sufficient oxygen by photosynthesis to surrounding cells and protect them against hypoxia. Thus, the cellular hypoxic conditions were firstly simulated by culturing human skin fibroblasts (HSFs) and human umbilical vein endothelial cells (HUVECs) under low-oxygen conditions (1% oxygen). A common hypoxic indicator {[Ru(dpp)_3_]Cl_2_} was utilized to detect intracellular hypoxia. It was found that the red fluorescence of HSFs incubated with MA-HF scaffolds in light was alleviated approximately to that in normoxia conditions, which is distinct from the large proportions of hypoxic cells in the HF, MA-HF (dark), and control groups (Figure 4(b)). Benefiting from the hypoxia alleviated by the photosynthetic scaffolds, the cell proliferation of HSFs incubated with MA-HF scaffolds in light was significantly promoted as compared to that of the other groups (Figures 4(c) and 4(d)). To explore whether the photosynthetic scaffolds could promote cell migration in hypoxic conditions, a typical scratch assay was conducted. The results revealed that the wound closure of both HSFs and HUVECs was significantly accelerated in the MA-HF (light) groups in comparison to the other groups (Figures 4(e) and 4(f) and Figure S11). Furthermore, the enhanced cell mobility by MA-HF scaffolds in light was also confirmed by the transwell migration assay of hypoxic HUVECs (Figures 4(g) and 4(h)). To evaluate the angiogenic capability of photosynthetic MA-HF scaffolds, a Matrigel tube formation assay was performed and the results showed that more vessel-like tubes were observed after 6 hours in the MA-HF (light) groups than those in other groups (Figures 4(i) and 4(j)). Taken together, these results indicated the symbiotic relationship between mammalian cells and microalgae-laden scaffolds, which is consistent with previous studies [20, 28]. More importantly, our photosynthetic MA-HF scaffolds could effectively reverse the cellular hypoxia and improve the biological functions of mammalian cells under hypoxic conditions.

**Figure 5:**
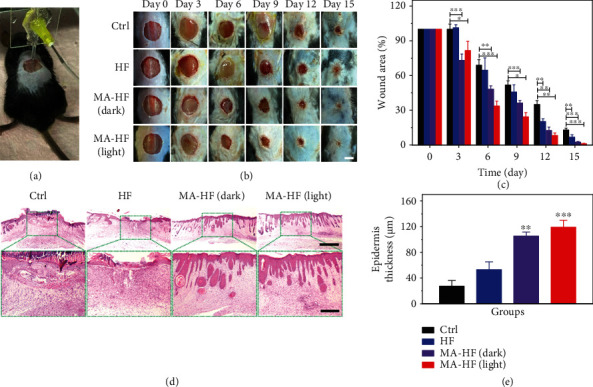
In situ bioprinting of MA-HF scaffolds for healing chronic wounds. (a) Representative photograph showing the MA-HF scaffold directly printed onto a murine diabetic wound via the microfluidic-driven printing strategy. (b) The wound healing process in different groups was tracked over 15 days. (c) Quantification of the in vivo wound closure at different time points. (d) Representative H&E staining images and (e) epidermis thickness of different groups on day 15. Dashed boxes indicate magnified regions. The scale bars indicate 5 mm in (b), 1 mm in (d, top), and 300 *μ*m in (d, bottom). ^∗^*p* < 0.05, ^∗∗^*p* < 0.01, and ^∗∗∗^*p* < 0.001 compared with control.

**Figure 6:**
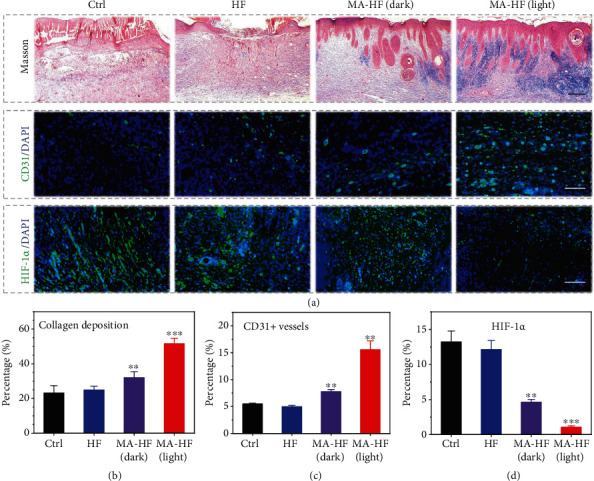
Accelerated collagen deposition, promoted angiogenesis, and alleviated tissue hypoxia by MA-HF scaffolds. (a) Masson's Trichrome staining (top) and immunofluorescence staining (CD31, middle; HIF-1*α*, bottom) images of the regenerated skin tissues on day 15. Quantification of the (b) collagen deposition, (c) CD31-positive microvessel densities, and (d) HIF-1*α* densities in different groups. All scale bars indicate 200 *μ*m in (a). ^∗∗^*p* < 0.01 and ^∗∗∗^*p* < 0.001 compared with control.

The in vivo wound healing potential of the photosynthetic MA-HF scaffolds was further investigated using a typical diabetic wound model regarding its chronic hypoxic features. Typically, the HF and MA-HF scaffolds were directly printed into the wounds on the dorsum of each mouse under anesthesia (Figure 5(a)). The healing process was tracked over 15 days, and the wounds healed faster with the treatment of MA-HF scaffolds as compared to the HF and control groups (Figure 5(b)). Quantitative analysis revealed that the relative wound area of the control, HF, MA-HF (dark), and MA-HF (light) groups on day 15 was 13.0 ± 1.5%, 6.8 ± 2.0%, 2.5 ± 0.5%, and 1.1 ± 0.3%, respectively (Figure 5(c)). Notably, the wound closure over the healing process did not show significant differences between the MA-HF (dark) and MA-HF (light) groups, indicating that the MA-HF scaffold exerted a stimulatory effect on wound healing even without light illumination. These results suggested that the degradable products of MA-HF scaffolds may be beneficial for wound healing, considering that the MA-HF scaffolds are degradable in a physiological microenvironment. Histological analysis was performed on day 15 with hematoxylin-eosin (H&E) staining and Masson's Trichrome staining. As shown in Figures 5(d) and 5(e), a thicker and more clear stratified epidermis layer with several appendages was formed in the two MA-HF groups, in dramatic contrast to the incomplete epidermis observed in the control and HF groups. Furthermore, the wounds treated with the MA-HF scaffold in light also showed the highest extents of collagen deposition (51.6 ± 3.0%), compared with those in the control (23.2 ± 4.2%), HF (24.9 ± 2.2%), and MA-HF (dark, 32.0 ± 3.4%) groups (Figures 6(a) and 6(b)), indicating the enhanced ECM reconstruction and tissue remodeling ability by the photosynthetic MA-HF scaffolds.

To explore the internal mechanism of the promoted wound closure by MA-HF scaffolds, immunohistochemical staining was performed using antibodies specific for CD31 (a typical marker of vascular endothelial cells) and HIF-1*α* (hypoxia inducible factor *α*, the main indicator of tissue hypoxia, Figures 6(a), 6(c), and 6(d)). With the treatment of the MA-HF scaffolds in light, the density of CD31-positive microvessels was significantly increased (15.6 ± 1.6%), while the average microvessel densities were 5.5 ± 0.1%, 5.0 ± 0.2%, and 7.8 ± 0.4% in the control, HF, and MA-HF (dark) groups, respectively (Figure 6(c)). Conversely, the relative expression level of HIF-1*α* in the MA-HF (light) group was 1.1 ± 0.2%, which was considerably less than that in the control (13.2 ± 1.5%), HF (12.1 ± 1.2%), and MA-HF (dark, 4.6 ± 0.4%) groups (Figure 6(d)). These findings suggested that the photosynthetic MA-HF scaffolds could effectively alleviate tissue hypoxia and promote angiogenesis in chronic wounds.

## 3. Conclusion

In summary, we have presented an in situ bioprinting strategy for fabricating the microalgae-laden hollow fibrous scaffolds with autotrophic oxygen-generating capability for adapting irregular-shaped wounds and promoting their healing. Owing to the oxygenic photosynthesis of the embedded living microalgae, the scaffolds could produce sustainable oxygen under light illumination, which facilitated cell proliferation, migration, and differentiation in low-oxygen culture conditions. Moreover, the living microalgae-laden scaffold could be directly printed into diabetic wounds and serve as an effective autotrophic biosystem to overcome the hypoxic microenvironment and accelerate the wound closure by increasing angiogenesis and promoting collagen synthesis. Therefore, our present work demonstrates the feasibility of in situ bioprinting of photosynthetic microalgae-laden scaffolds for autotrophic wound healing, which also provides an insightful therapeutic strategy for diverse tissue engineering applications. To adapt to irregular, curved, or deep wounds in complex biological environments, further optimization of microfluidic-assisted bioprinting is needed to comprehensively demonstrate the efficacy. Our current printing system defined the scaffold geometry before printing. For real-time bioprinting, the intraoperative computerized imaging systems can be utilized to construct the real-time tomography of the tissue defects in the future. Moreover, the advances in microfluidic chips and functional materials with better bioprinting compatible properties will enable the microfluidic-assisted bioprinting to print more complicated 3D architectures into the curvilinear and deep tissue defects. To this end, we believe that our in situ bioprinting system will provide a facile and versatile strategy for repairing diverse defects in a rapid, safe, and automated manner.

## 4. Materials and Methods

### 4.1. Materials

Sodium alginate was purchased from Alfa Aesar. Methacrylate gelatin (GelMA), lithium phenyl-2,4,6-trimethylbenzoylphosphinate (LAP), and calcium chloride (CaCl_2_) were bought from Shanghai Aladdin. Tris (4,7-diphenyl-1,10-phenanthroline) ruthenium (II) dichloride {[Ru(dpp)_3_]Cl_2_} was obtained from Shanghai Macklin. The microalgae (MA, *Chlorella pyrenoidosa*) and the specialized microalgae culture medium were purchased from Nanjing Health Biotech. Ultrapure water (18.2 M*Ω*·cm^−1^, Millipore) was used throughout the experiments.

### 4.2. Microfluidic Spinning of Microalgae-Laden Hollow Fibers

The microalgae-laden hollow fibers (MA-HF) were prepared using a coaxial microfluidic chip assembled from a piece of glass slide and two cylindrical glass capillaries. In a typical experiment, to generate the hydrogel fibers with hollow channels, a capillary with a spindle tip (orifice diameter: 100 *μ*m) was coaxially inserted into a capillary with a tapered tip (orifice diameter: 450 *μ*m, Figure S1), which were then glued to a glass slide with transparent epoxy resin. The outer phase was pregel aqueous solution containing GelMA (5% *w*/*v*), sodium alginate (2.5% *w*/*v*), and LAP (0.1% *v*/*v*). The inner phase was gelatin solution (5% *w*/*v*) containing various CaCl_2_ contents (0~2% *w*/*v*). Both the inner and outer liquids were injected into the microfluidic chip using programmed syringe pumps with defined flow rates and then collected in a clean petri dish. The hollow fibers were firstly generated by the ion crosslinking reaction between Ca^2+^ and alginate and subsequent photopolymerization of the GelMA component under ultraviolet (UV, 365 nm, 100 W) light irradiation for 5 min. A series of microalgae-laden hollow fibers with various concentrations could be fabricated by varying the MA contents in the pregel solutions (MA content: 1, 5, 10, 50, or 100 × 10^5^ cells/mL).

### 4.3. In Situ Bioprinting of Microalgae-Laden Hollow Fibrous Scaffolds

Microalgae-laden hollow fibrous scaffolds (MA-HF scaffolds) were in situ printed in a dry petri dish through the microfluidic-assisted bioprinting strategy. The custom-made capillary microfluidic chip was used to replace the original 3D printer nozzle. The scaffold geometry (sizes and shapes) for printing was designed using 3ds max 2020 software. The extrusion rate of the microfibers from the microfluidic chip should match the moving speed of 3D printing platform for a fluent printing process. Typically, both the inner and outer flow rates were set at 2 mL/h, and the moving speed of the 3D printer was set at 5 mm/s. During printing, the ion crosslinking of the alginate component in the outer flow phase was initially induced by the diffusion of Ca ions from the inner flow phase. After printing, the crosslinking of the GelMA component was achieved by UV irradiation (365 nm, 100 W, 5 min). All liquids and reagents used for subsequent biological experiments were sterilized by filtration through a sterile 0.22 mm filter or exposure to UV irradiation (254 nm) overnight.

### 4.4. Characterizations

Optical bright-field and fluorescent images of the hollow microfibers and scaffolds (containing green fluorescent nanoparticles of 501/515 nm) were observed by a stereomicroscope (Olympus BX51, Tokyo, Japan). The microstructure and morphology of the freeze-dried microalgae and microalgae-laden fibrous scaffolds were characterized with scanning electron microscopy (SEM, SU8010, Hitachi, Japan).

### 4.5. Dissolved Oxygen Release from the Microalgae-Laden Hollow Scaffolds

The photosynthetic oxygen production capacity of MA-HF scaffolds (MA concentration: 10^6^ cells/mL; size: 15 mm × 15 mm × 2 mm) was examined under the illumination of a LED light bulb. In this study, the light intensity was set at 6000 lux, because the optimum light regimes for the growth of *Chlorella pyrenoidosa* are under the light intensity of 5000 to 8000 lux according to the manufacturer's instructions. The distance between the scaffolds and the LED light was 10 cm. The growth of the microalgae within the MA-HF scaffolds was photographed every two days at 25°C. The photoautotrophic oxygen release from MA-HF scaffolds was recorded in real time using an oxygen microsensor under the light illumination at 25°C and/or 37°C. To test the controllable oxygen consumption and production, the MA-HF scaffold was exposed to the LED light (light on) for 60 min, followed by incubation in dark conditions (light off) for another 60 min. The 60 min light on/off cycle was repeated for 10 h.

### 4.6. In Vitro Degradation Behavior of the Microalgae-Laden Hollow Scaffolds

The initial weights of MA-HF scaffolds (MA concentration: 10^6^ cells/mL; size: 20 mm × 20 mm × 3 mm) were weighed after being soaked in 2% CaCl_2_ for 24 h. The scaffold was immersed in 2 mL of phosphate-buffered saline (PBS) and kept in dark at 37°C for 8 days. The scaffolds were photographed every day and weighed after wiping out water on the scaffold surface. The relative weight loss was calculated as follows: relative weight loss (%) = (*W*_0_ − *W*_*t*_)/*W*_0_ × 100. Here, *W*_*t*_ represented the scaffold weights at certain time points and *W*_0_ represented initial weights.

### 4.7. Cell Culture

Human skin fibroblasts (HSFs, ATCC) were cultured in Dulbecco's modified eagle medium (DMEM) containing 10% of fetal bovine serum and 1% of penicillin-streptomycin double antibiotics, while human umbilical vein endothelial cells (HUVECs, ScienCell) were cultured in the specified endothelial cell medium (ECM) in 5% CO_2_ at 37°C.

### 4.8. Intracellular Hypoxic Alleviation

The intracellular hypoxic alleviation effect of MA-HF scaffolds was evaluated using a hypoxia indicator {[Ru(dpp)_3_]Cl_2_} with red fluorescence, which could be quenched by oxygen. HSFs were incubated in a 24-well plate (10^5^ cells/well) under normoxia for 12 h. For hypoxic groups, the cells were cultured in the hypoxic microenvironment (1% O_2_) and the transwell inserts (0.4 *μ*m pore-sized filters) loaded with MA-HF scaffolds (1 × 10^6^ cells/mL) or HF scaffolds (*Φ* 5 mm × 2 mm) were gently placed into the 24-well plate. The MA-HF scaffolds in the MA-HF (light) groups were then illuminated by the LED light (6000 lux) for 6 h. After another 6 h in dark, the MA-HF scaffolds were removed and the cell medium was replaced with fresh DMEM containing hypoxia probes (8 *μ*g/mL). The probe solution was discarded after 4 h incubation at 37°C. The cells were washed with fresh DMEM and fixed in paraformaldehyde (4%). 4′,6-Diamidino-2-phenylindole (DAPI, blue) was used to stain the cell nuclei for observation.

### 4.9. Cell Proliferation Assay

HSFs were cultured in a 24-well plate (1 × 10^4^ cells/well) for overnight. Transwell inserts (0.4 *μ*m pore-sized filters) containing MA-HF scaffolds (1 × 10^6^ cells/mL) or HF scaffolds (*Φ* 5 mm × 2 mm) were gently transformed into the plate. The MA-HF scaffolds in the MA-HF (light) groups were illuminated by the LED light (6000 lux) for 6 h every day. The cells were incubated in the hypoxic microenvironment (1% O_2_) with the scaffolds for 5 days, and their proliferative status was examined by CCK-8 assay. Additionally, the cell viability of the HSFs was visualized with double staining of Calcein-AM and PI.

### 4.10. Scratch Wound Healing Assay

HSFs or HUVECs were seeded in a 24-well plate (1 × 10^5^ cells/well in the lower chamber) and cultured in the hypoxic microenvironment (1% O_2_). After 12 h, a sterile p200 pipette tip was used to scratch the single-layer cells. The unattached cells were rinsed with PBS twice. Transwell inserts (0.4 *μ*m pore-sized filters) containing MA-HF scaffolds (1 × 10^6^ cells/mL) or HF scaffolds (*Φ* 5 mm × 2 mm) were gently transformed into the plate (in the upper chamber). The MA-HF scaffolds in the MA-HF (light) groups were continuously exposed to the LED light (6000 lux). The cells in the lower chambers were photographed at appropriate time points. The relative migration area was defined as: relative migration area (%) = (1 − *W*/*W*_0_) × 100%. *W* represented the wound area at certain time points and *W*_0_ represented the wound area immediately after scratching, which were acquired using ImageJ software.

### 4.11. Transwell Migration Assay

HUVECs were cultured in the transwell inserts with 8 *μ*m pore-sized filters (1 × 10^4^ cells/well in the upper chamber) and incubated in a 24-well plate containing MA-HF scaffolds (1 × 10^6^ cells/mL, *Φ* 10 mm × 1 mm) or HF scaffolds (*Φ* 10 mm × 1 mm in the lower chamber) in 1% oxygen for 24 h. The MA-HF scaffolds in the MA-HF (light) groups were continuously exposed to the LED light (6000 lux). Cotton swabs were used to remove the cells on the upper surface of transwell inserts. Crystal violet solution (0.1%) was used to stain the migrated cells to the bottom side of the filters. The cells in purple were photographed by an inverted optical microscope (Nikon, Japan).

### 4.12. Tube Formation Assay

Matrigel matrix (BD, USA) was used to coat a 24-well plate (250 *μ*L per well) before the cell seeding of HUVECs (5 × 10^4^ cells/well). The transwell inserts loaded with MA-HF scaffolds (1 × 10^6^ cells/mL) and HF scaffolds (*Φ* 5 mm × 2 mm) were gently placed into the 24-well plate and kept in 1% oxygen under the LED light illumination (6000 lux) for 6 h. The tube formation of HUVECs was observed using a fluorescence microscope (Zeiss) after Calcein-AM staining.

### 4.13. In Vivo Chronic Wound Closure in Diabetic Mice

The male C57BL/6 mice (20~25 g, 7~8 weeks) were provided by Beijing Vital River Laboratory Animal Technology Co., Ltd. All rats were treated strictly according to the Laboratory Animal Care and Use Guidelines. The experimental protocol was approved by the Animal Care and Use Committee of Wenzhou Medical University (Zhejiang, China). Streptozotocin- (STZ-) induced diabetic mouse model was established by intraperitoneal injection of STZ (50 mg/kg, dissolved in 0.1 M citrate buffer, pH~4.5) into the mice. Four weeks later, blood glucose from tail blood was measured and the diabetic mice with blood glucose levels over 20 mM were selected for further experiments. The mice were randomly grouped as follows (*n* = 8): (1) control group, (2) HF group, (3) MA-HF (dark) group, and (4) MA-HF (light) group. A full-thickness wound (*Φ* 10 mm) was created on the shaved dorsum of each mouse, which was rapidly covered by the in situ bioprinting of HF or MA-HF scaffolds (*Φ* 10 mm × 2 mm). During printing, the progels were firstly polymerized by the ion crosslinking between the alginate component and Ca ions within 2 min. Immediately after printing, 1 min UV irradiation was performed to polymerize the GelMA component in the progels. The scaffolds were then shielded with an opaque adhesive bandage. The MA-HF scaffolds in the MA-HF (light) groups were exposed to the LED light (6000 lux) for 2 hours every three days. The skin wound areas were recorded using a digital camera and calculated as: relative wound area (%) = *W*_*t*_/*W*_0_ × 100%. Here, *W*_*t*_ represented the wound area on day *t* (*t* = 0, 3, 6, 9, 12, and 15) and *W*_0_ represented the wound area immediately after wounding, which were acquired using ImageJ software. All mice were sacrificed and sampled on day 15. For histological analysis, typical hematoxylin-eosin (H&E) staining and Masson's Trichrome staining were performed. Moreover, immunohistochemical analysis of CD31 and HIF-1*α* was employed to evaluate the vascularity and tissue hypoxia in the regenerated skin tissues. The tissue slices (thickness: 5 *μ*m) were incubated with anti-CD31 or HIF-1*α* antibodies. The cell nuclei were stained with DAPI. Both the CD31-positive vessels (green), HIF-1*α* protein (green), and nuclei (blue) were observed under a fluorescence microscope (Zeiss).

### 4.14. Statistical Analysis

Data are expressed as means ± standard deviations (*n* ≥ 4). All graphs were created from OriginPro 2020 software. The statistical significance between two groups was calculated using two-tailed unpaired Student's *t*-tests, with a *p* value < 0.05 considered significant (^∗^*p* < 0.05, ^∗∗^*p* < 0.01, and ^∗∗∗^*p* < 0.001).

## Data Availability

All data needed to evaluate the conclusions in the paper are present in the paper and/or the Supplementary Materials. Additional data related to this paper may be requested from the authors.

## Supplementary Materials

Supplementary Materials Figure S1: the photographs and optical images of the coaxial capillary microfluidic device. Figure S2: the optical images of hollow fibers generated from the microfluidic device at varied CaCl_2_ concentrations or the inner/outer flow rates. Figure S3: the optical and fluorescent micrographs of the hollow fibers. Figure S4: the digital photographs of the continuous pure hollow fibers and microalgae-loaded hollow fibers. Figure S5: the digital photographs of the printed scaffolds with different shapes and sizes. Figure S6: the SEM images of the microalgae incorporated into the MA-HF scaffolds. Figure S7: the SEM images of the freeze-dried HF scaffolds at different magnifications. Figure S8: the SEM images of the microalgae at different magnifications. Figure S9: the oxygen production of MA-HF scaffolds under light illumination for 7 days. Figure S10: the in vitro degradation behavior of MA-HF scaffolds. Figure S11: the in vitro scratch assay of HUVECs cultured with HF or MA-HF scaffolds.
