# Supplementary material for: In Situ 3D Bioprinting Living Photosynthetic Scaffolds for Autotrophic Wound Healing
Source: Research (Wash D C). 2022 Mar 20;2022:9794745. doi: 10.34133/2022/9794745 (PMC8961369; doi:10.34133/2022/9794745)
Supplement: Supplementary Materials — Figure S1: the photographs and optical images of the coaxial capillary microfluidic device. Figure S2: the optical images of hollow fibers generated from the microfluidic device at varied CaCl2 concentrations or the inner/outer flow rates. Figure S3: the optical and fluorescent micrographs of the hollow fibers. Figure S4: the digital photographs of the continuous pure hollow fibers and microalgae-loaded hollow fibers. Figure S5: the digital photographs of the printed scaffolds with different shapes and sizes. Figure S6: the SEM images of the microalgae incorporated into the MA-HF scaffolds. Figure S7: the SEM images of the freeze-dried HF scaffolds at different magnifications. Figure S8: the SEM images of the microalgae at different magnifications. Figure S9: the oxygen production of MA-HF scaffolds under light illumination for 7 days. Figure S10: the in vitro degradation behavior of MA-HF scaffolds. Figure S11: the in vitro scratch assay of HUVECs cultured with HF or MA-HF scaffolds. [file 9794745.f1.docx]

**Supporting Information**

**In Situ 3D-bioprinting Living Photosynthetic Scaffolds for Autotrophic Wound Healing**

Xiaocheng Wang^1,2,3^, Chaoyu Yang^2,3^, Yunru Yu^2,3,4^, Yuanjin Zhao^1,2,3,4,^*

1. Department of Rheumatology and Immunology, Institute of Translational Medicine, The Affiliated Drum Tower Hospital of Nanjing University Medical School, Nanjing 210008, China

2. Oujiang Laboratory (Zhejiang Lab for Regenerative Medicine, Vision and Brain Health), Wenzhou, Zhejiang 325001, China

3. Wenzhou Institute, University of Chinese Academy of Sciences, Wenzhou 325001, China

4. State Key Laboratory of Bioelectronics, School of Biological Science and Medical Engineering, Southeast University, Nanjing 210096, China

* Email: [yjzhao@seu.edu.cn](mailto:yjzhao@seu.edu.cn).


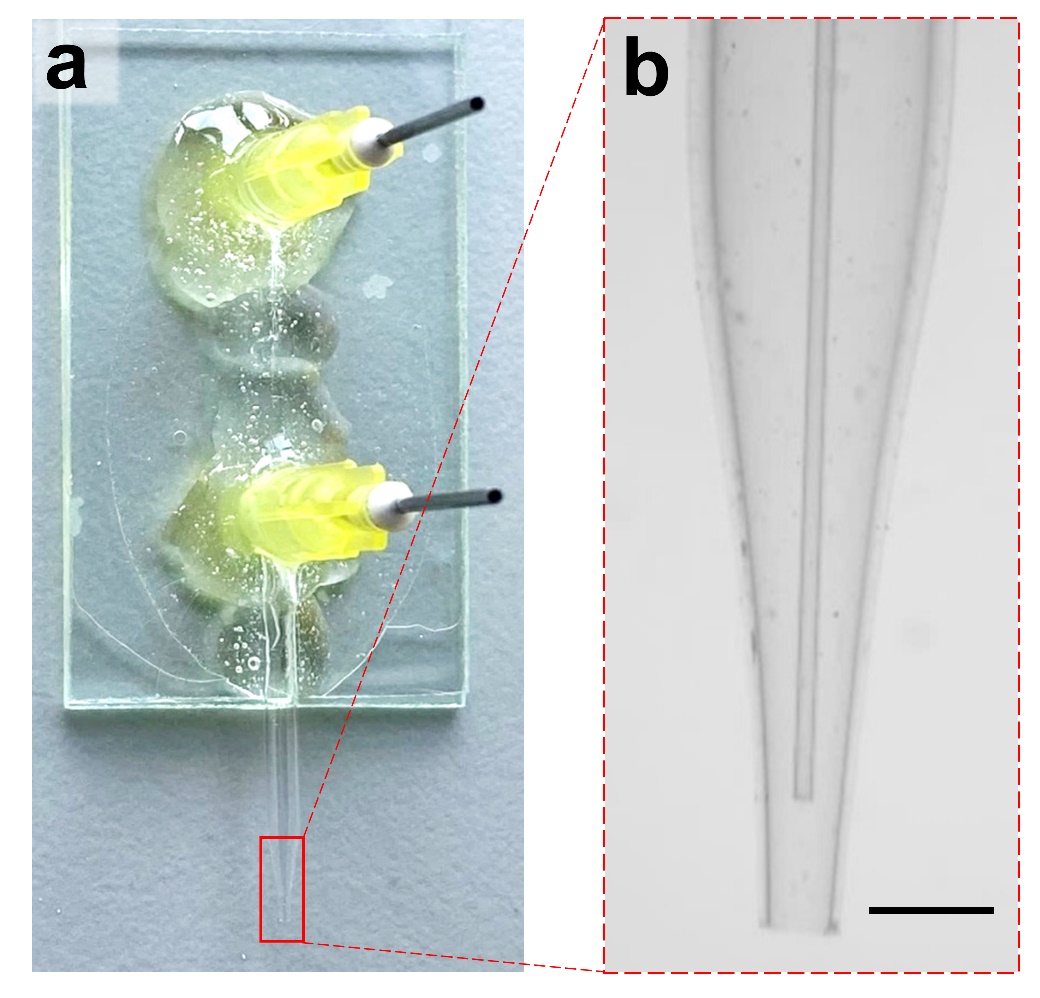


**Figure S1.** (a) Digital photographs of the capillary microfluidic device and (b) their coaxial microstructure for the generation of the hollow fibers. Scale bar, 1 mm.


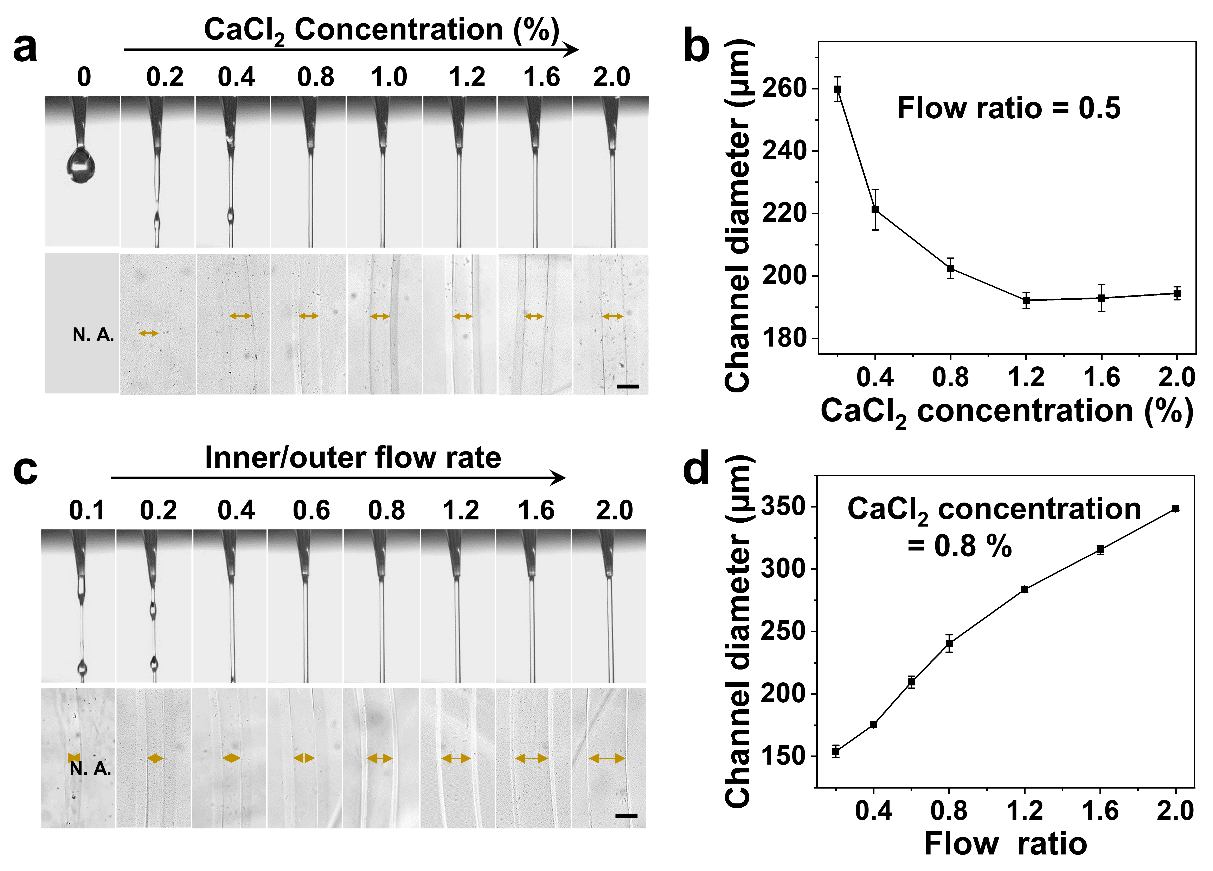


**Figure S2.** (a, c) Optical images of microfluidic spinning hollow fibers with straight micro-channels. The brown arrows indicate the channel diameters. N.A. indicates not available. Scale bar, 200 μm. Relationships between the channel diameter of the hollow fibers and (b) CaCl_2_ concentration or (d) the inner/outer flow rate.


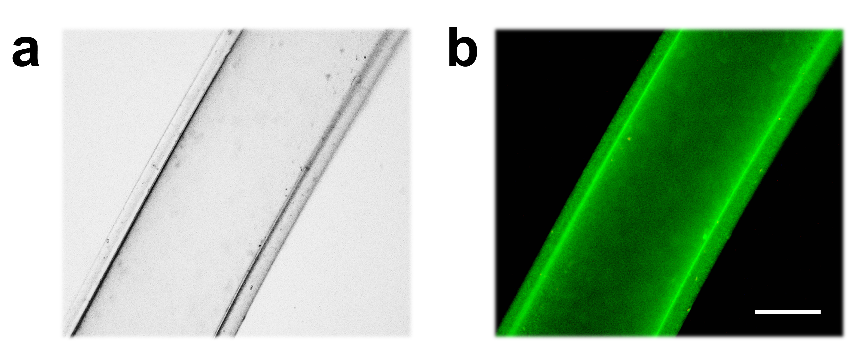


**Figure S3.** (a) Optical and (b) fluorescent micrographs of the hollow fibers containing green fluorescent nanoparticles of 501/515 nm. Scale bar, 150 μm.


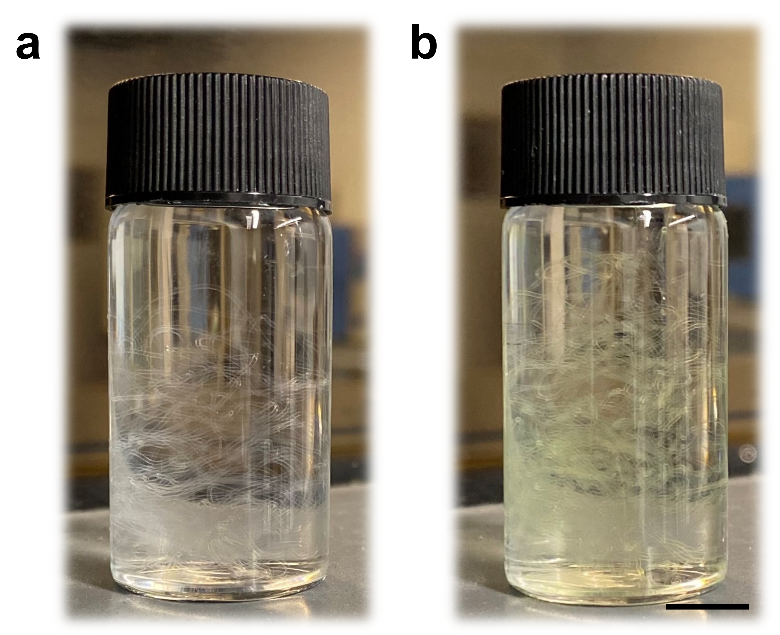


**Figure S4.** Digital photographs of the continuous (a) pure hollow fibers and (b) microalgae-loaded hollow fibers (10^7^ cells/mL) in glass vials. Scale bar, 1 cm.


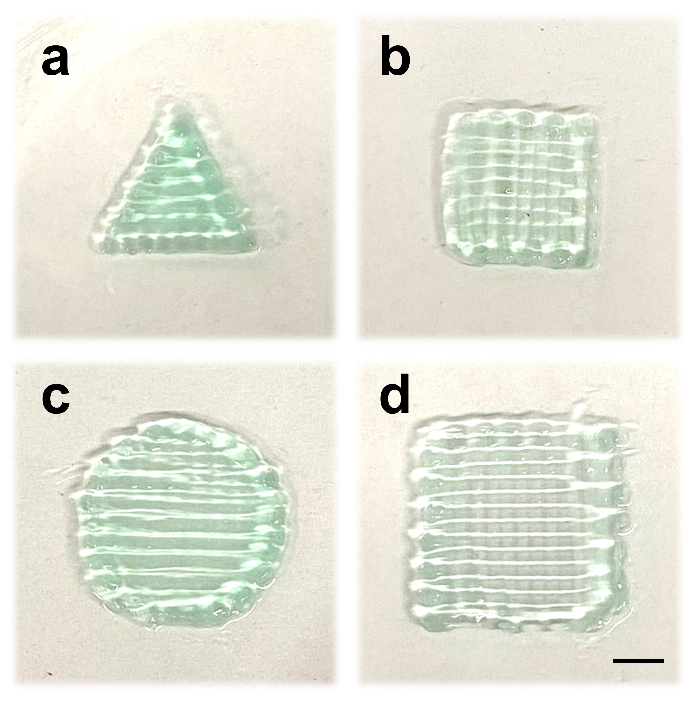


**Figure S5.** Digital photographs of the printed scaffolds with different shapes and sizes. Scale bar, 5 mm.


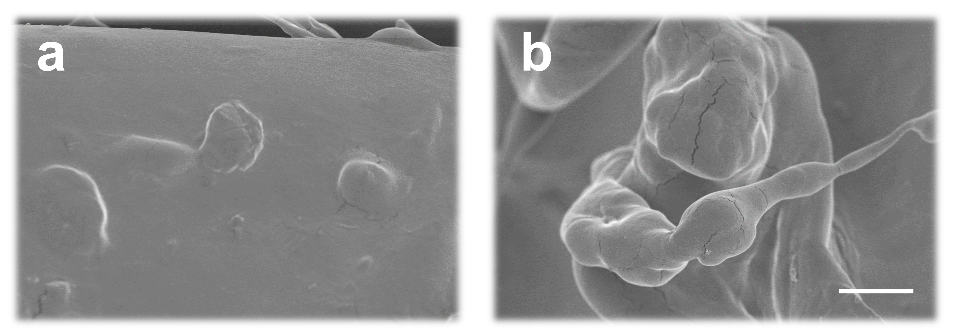


**Figure S6.** SEM images showing the microalgae incorporated into the MX-HF scaffold matrix. The scale bar indicates 5 μm.


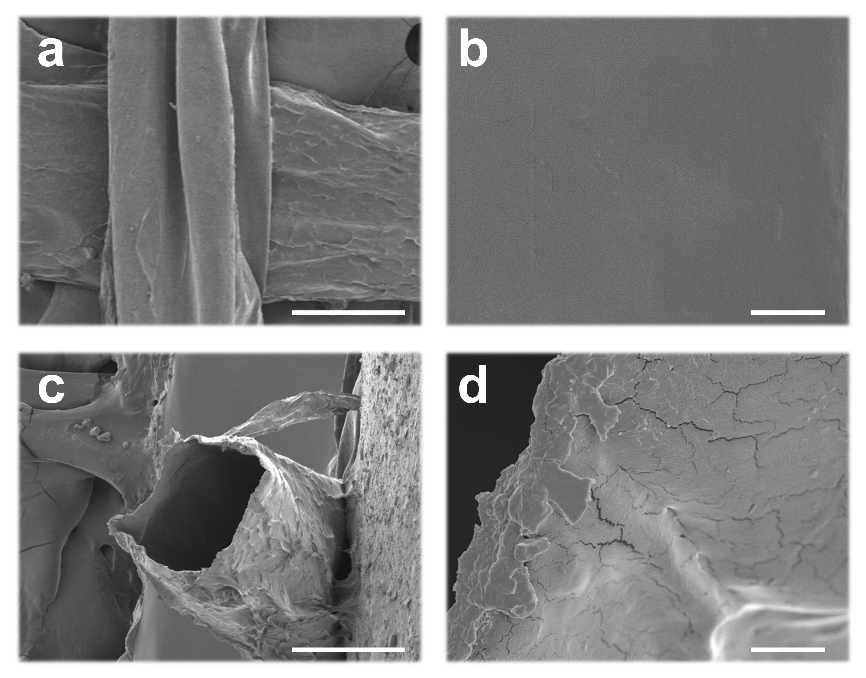


**Figure S7.** (a, b) Top and (c, d) section views of the scanning electron microscope (SEM) images of the freeze-dried HF scaffolds at different magnifications. The scale bars indicate 200 μm in (a, c) and 5 μm in (b, d).


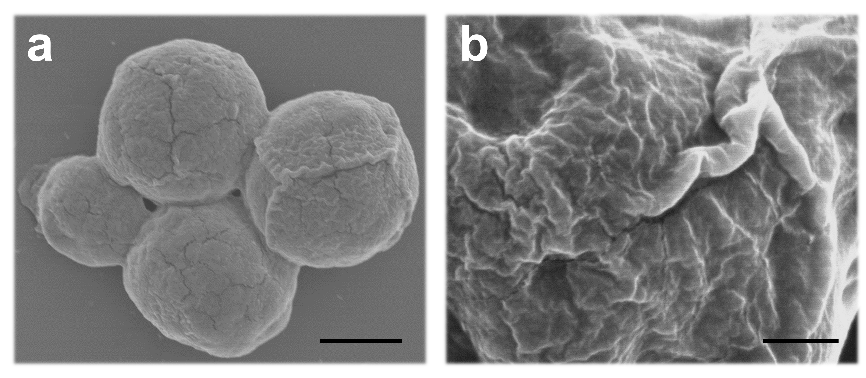


**Figure S8.** SEM images of the microalgae at different magnifications. The scale bars indicate 2 μm in (a) and 300 nm in (b).


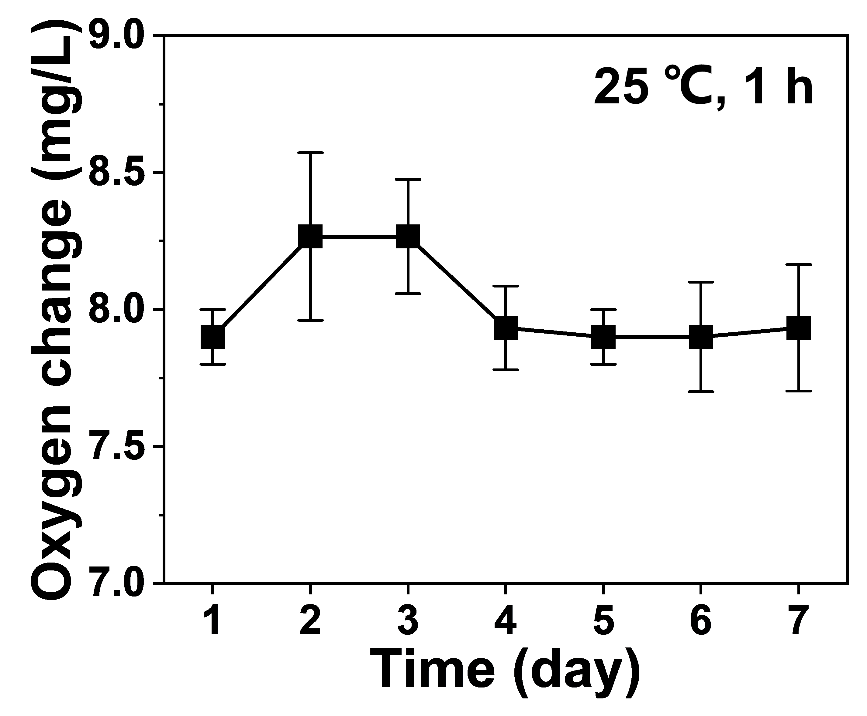


**Figure S9.** Oxygen production of MA-HF scaffolds (1 × 10^6^ cells/mL) under light illumination (6000 lux) for 60 min at 25 °C after storage at 4 °C for 7 days.


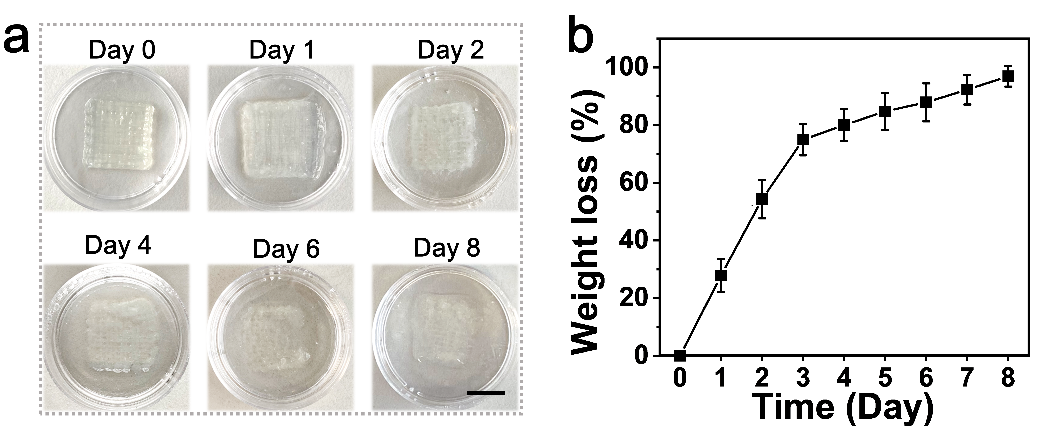


**Figure S10.** (a) Representative photographs of the MA-HF scaffolds immersed in PBS in dark at 37 ℃ for 8 days. (b) Quantitative analysis of weight loss at different time points. The scale bar indicates 1 cm in (a).


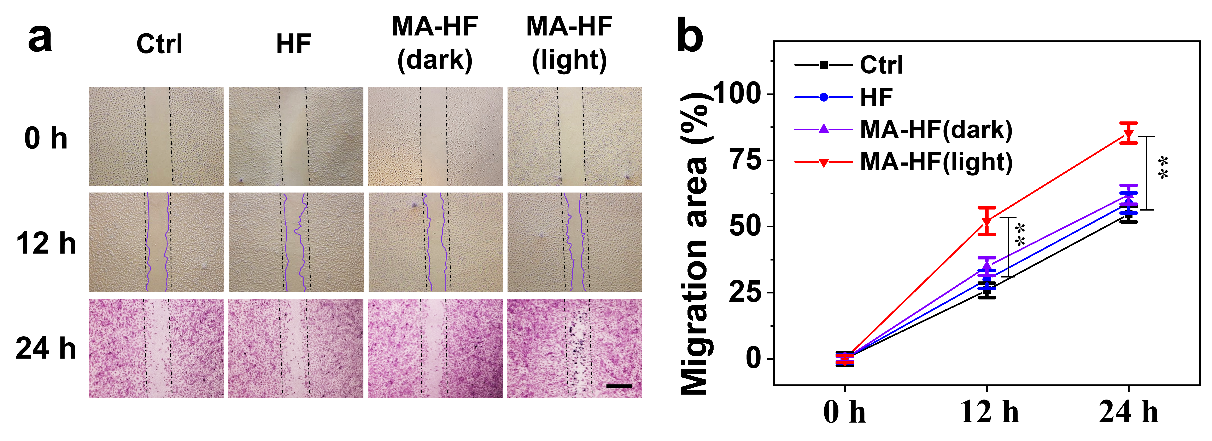


**Figure S11.** (a) In vitro scratch assay of human umbilical vein endothelial cells (HUVECs) cultured with HF, MA-HF scaffolds under dark or light conditions. (b) Quantification of the relative wound area (**p < 0.01 compared with control). The scale bars indicate 500 μm in (a).
